# A Conserved Cysteine Residue of *Bacillus subtilis* SpoIIIJ Is Important for Endospore Development

**DOI:** 10.1371/journal.pone.0099811

**Published:** 2014-08-18

**Authors:** Luísa Côrte, Filipa Valente, Mónica Serrano, Cláudio M. Gomes, Charles P. Moran, Adriano O. Henriques

**Affiliations:** 1 Instituto de Tecnologia Química e Biológica, Universidade Nova de Lisboa, Oeiras, Portugal; 2 Department of Microbiology and Immunology, Emory University School of Medicine, Atlanta, Georgia, United States of America; University of Groningen, Groningen Institute for Biomolecular Sciences and Biotechnology, Netherlands

## Abstract

During sporulation in *Bacillus subtilis*, the onset of activity of the late forespore-specific sigma factor σ^G^ coincides with completion of forespore engulfment by the mother cell. At this stage, the forespore becomes a free protoplast, surrounded by the mother cell cytoplasm and separated from it by two membranes that derive from the asymmetric division septum. Continued gene expression in the forespore, isolated from the surrounding medium, relies on the SpoIIIA-SpoIIQ secretion system assembled from proteins synthesised both in the mother cell and in the forespore. The membrane protein insertase SpoIIIJ, of the YidC/Oxa1/Alb3 family, is involved in the assembly of the SpoIIIA-SpoIIQ complex. Here we show that SpoIIIJ exists as a mixture of monomers and dimers stabilised by a disulphide bond. We show that residue Cys134 within transmembrane segment 2 (TM2) of SpoIIIJ is important to stabilise the protein in the dimeric form. Labelling of Cys134 with a Cys-reactive reagent could only be achieved under stringent conditions, suggesting a tight association at least in part through TM2, between monomers in the membrane. Substitution of Cys134 by an Ala results in accumulation of the monomer, and reduces SpoIIIJ function *in vivo*. Therefore, SpoIIIJ activity *in vivo* appears to require dimer formation.

## Introduction

Protein secretion and membrane protein insertion are fundamental processes in all living organisms, and several pathways are known to serve those purposes, namely the Sec, Tat, and YidC/Oxa1/Alb3 pathways [1], [2], [3]. These pathways have been extensively studied in bacteria, specifically in the model organisms *Escherichia coli* and *Bacillus subtilis*. The most prominent one is the Sec pathway. Its core is the SecYEG channel, which is utilised both for secretion and membrane protein insertion, using distinct piloting factors that guide each class of proteins to SecYEG. The SecB chaperone in *E. coli* (possibly CsaA in *B. subtilis*) guides proteins to be secreted. In contrast, nascent membrane proteins are delivered to SecYEG by the signal recognition particle (SRP) [reviewed in 1], [4]. The twin-arginine translocation (Tat) pathway has the peculiarity of permitting the transport of previously folded proteins [reviewed in 2]. YidC/Oxa1/Alb3 family members, found in archaea, bacteria and in eukaryotic organelles, participate in the biogenesis of membrane proteins. The first protein from this family to be identified was Oxa1 in mitochondria of *Saccharomyces cerevisiae*. Members of this family are polytopic membrane proteins that share a core architecture of five transmembrane (TM) segments. They perform critical roles in membrane insertion and folding and also in the assembly of energy-transducing multimeric membrane complexes [reviewed in 3]. The best-studied proteins from this family in bacteria are YidC (*E. coli*) and SpoIIIJ (*B. subtilis*), respectively, which function with the Sec channel or independently of it. The Sec-independent function of these proteins is conserved and similar to that of Oxa1 in mitochondria, which lack the Sec system [3], [5], [6]. The large periplasmic region of YidC (absent in SpoIIIJ and from the conserved core that characterises the family) was found to be monomeric [7], [8]. However, full-length YidC was observed as a dimer through cryo-electron microscopy [9], suggesting that the determinants for dimerisation lie within the conserved, membrane-associated, regions of the protein. SpoIIIJ is required for spore formation in *B. subtilis* [10]. The conversion of vegetative cells into highly resistant spores is one of the responses that this organism uses to cope with highly stressful situations [11], [12], [13]. Besides SpoIIIJ, another member of this family exists in *B. subtilis*, YqjG. Although they can substitute for each other during growth, only SpoIIIJ supports efficient sporulation [10], [14], [15], [16], [17].

Spore differentiation takes place in a cell divided into two unequally-sized compartments, between which successive waves of gene expression in one compartment or the other are activated in a coordinated manner and in register with the course of morphogenesis [11], [12], [13]. Transcription is controlled by the successive activities of cell type-specific RNA polymerase sigma factors. From the forespore, σ^F^ signals the activation of σ^E^ in the larger mother cell, which in turn is required for activity of σ^G^ in the forespore following engulfment completion. σ^G^ is in turn responsible for the activation of σ^K^ in the mother cell [18], [19]. A key process of endosporulation is the engulfment of the forespore by the mother cell. The onset of σ^G^ activity is normally coupled to engulfment completion, when the forespore becomes isolated from the extracellular milieu and surrounded by two membranes [13], [19]. At this stage of development, assembly of a specialised secretion system that spans the two forespore membranes is thought to allow the mother cell to feed the forming spore, allowing the continuation of macromolecular synthesis [10], [20], [21], [22], [23], [24]. Eight mother cell-specific proteins encoded by the *spoIIIA* operon, and the forespore-specific SpoIIQ protein form the complex. SpoIIIJ is needed specifically during this step of sporulation possibly because it participates in the biogenesis of the secretion complex [10], [20], [23], [24], [25], [26]. Evidence suggests that SpoIIIJ interacts with one of the *spoIIIA* proteins, SpoIIIAE, in the context of the Sec translocon to promote its correct insertion into the forespore membranes [20], [24].

Here we have purified SpoIIIJ from *E. coli* cells and shown that it forms a dimer. We also provide evidence that a cysteine residue predicted to be in the second transmembrane segment of SpoIIIJ facilitates dimer formation by forming a disulphide bond at the dimer interface. Replacement of the cysteine by an alanine residue results in accumulation of the monomer and under certain conditions impairs the activity of σ^G^ and reduces sporulation. Our results suggest a model in which a disulphide-bond contributes, with additional non-covalent interactions, to the formation of a SpoIIIJ dimer, and that this dimer is important for SpoIIIJ activity during sporulation.

## Materials and Methods

### Media, bacterial strains and general techniques

The *B. subtilis* strains used in this work are congenic derivatives of the Spo^+^ strain MB24 (*trpC2 metC3*) and are listed in Table S1. Luria-Bertani (LB) medium was used for growth or maintenance of *E. coli* and *B. subtilis*, and sporulation was induced in Difco sporulation medium (DSM) [27], [28].

### Strains and plasmids

A *spoIIIJ* ^C134A^ allele for expression in *E. coli* was constructed in two steps: an initial amplification from chromosomal DNA of the wild-type *B. subtilis* strain MB24 using primers pairs J112D with JC134A_D, and JC134A_R with Jhis (Table S2); next, the PCR fragments were joined using the external primers through splicing by overlap extension (SOE) [29]. The final PCR product was cleaved with *Nco*I and *Eco*RI, ligated to pETDuet-1 (Novagen, Darmstadt, Germany) similarly digested to obtain pFiV1 (plasmids are listed in Table S3), resulting in a fusion to a His_6_ tag at the 3′ of *spoIIIJ* ^C134A^.

For controlled expression of His_6_ fusions to *spoIIIJ* and *spoIIIJ* ^C134A^ in *B. subtilis* from a promoter that can be induced with isopropyl-β-D-thiogalactopyranoside (IPTG), chromosomal DNA of MB24 was amplified with primers J174D and JhisR (for wild-type *spoIIIJ*) and primer pairs J174D with JC134A_R, and JC134A_D with JhisR, being the last two products joined by PCR (SOE) with the external primers (for the *spoIIIJ* ^C134A^ mutant). The final PCR products were digested with *Bgl*II and *Sph*I and ligated to pDH88 [30] similarly digested, generating pFiV2 and pFiV3, respectively. Both pFiV2 and pFiV3 were digested with *Eco*RI and *Bam*HI, the inserts were recovered and ligated to pDG1664 [31] similarly digested to produce pFiV4 and pFiV5, respectively; these plasmids were inserted at the non-essential locus *thrC* of JOB44 [16] through a double recombination event, producing AH5425 (Δ*spoIIIJ* Δ*thrC*::P*_spac_-spoIIIJ- his*_6_) and AH5426 (Δ*spoIIIJ* Δ*thrC*::P*_spac_-spoIIIJ* ^C134A^ *-his_6_*, respectively. The two strains were transformed with Δ*yycR*::P*_sspE_-cfp* from AH6566 [24] (constructed with DNA from BTD2633 (kindly provided by D. Rudner) whilst selecting for Cm^r^, resulting in AH5431 and AH5432, respectively (Table S1). JOB44 was transformed with DNA of BTD2633 resulting in AH5433 (Δ*spoIIIJ* Δ*yycR*::P*_sspE_-cfp*).

To construct pLC138, two PCR products were obtained with primer pairs PYqjG-460D with YA50C_R, and YA50C_D with YqjG-His-R, which were joined by PCR (SOE) using the external primers. Both the final PCR product and pLC111 (Côrte and Henriques, unpublished data) were digested with *Bam*HI and ligated. To construct the *yqjG* ^A50C/C142A^-*his_6_* derivative, primers PYqjG-460D with YC142A_R were used to amplify the first half of the gene from pLC138, and primers YC142A_D with YqjG-His-R for the second half, using pLC115 (Côrte and Henriques, unpublished data) as a template. The products were joined by PCR (SOE) with the external primers, digested with *Bam*HI and cloned into pLC111 similarly digested, resulting in pLC155. Transformation of JOB44 with pLC155 resulted in AH5382 (Δ*spoIIIJ* Δ*amyE*::P*_yqjG_-yqjG* ^A50C/C142A^-*his_6_*).

### Production of SpoIIIJ-His_6_ and of SpoIIIJ^C134A^-His_6_

*E. coli* strain C43(DE3) bearing pMS266 or pFiV1, were grown in LB and induced with IPTG for 3 hours at 37°C [32]. Cells were broken in a French pressure cell (19,000 lb/in^2^) in lysis buffer [20 mM Tris-HCl pH 7.6, 50 mM NaCl, 20% glycerol, 1 mM phenylmethylsulphonyl fluoride (PMSF), DNAse I].

The whole-cell extract was separated in a soluble and a membrane fraction by ultracentrifugation (100,000× *g*, 45 min, at 4°C). Membrane proteins were extracted from the pellet with 2% dodecyl maltoside (DDM), 20 mM Tris-HCl pH 7.6, 20% glycerol, 0.5 M NaCl and 10 mM imidazole, for 1 h on ice, and again ultracentrifuged (as above). Purification proceeded using a Ni^2+^-NTA affinity column (Qiagen, Hilden, Germany) previously equilibrated with a buffer composed of 0.5 M NaCl, 0.1% DDM 20 mM Tris-HCl pH 8 with 20 mM imidazole. The column was washed five times with the same buffer. Bound proteins were was eluted with a similar buffer containing 25 mM, 50 mM or 100 mM imidazole.

### Circular dichroism

Purified SpoIIIJ-His_6_ or SpoIIIJ^C134A^-His_6_ were first dialysed in 20 mM Tris-HCl pH 7.6, 10% glycerol, 0.5 M NaCl, 0.1% DDM. Far-UV CD spectra were measured at 20°C on a Jasco J-815D CD spectrometer using a quartz polarised 1 mm path length cuvette, from 200 to 260 nm.

### Size exclusion chromatography

SpoIIIJ-His_6_ or SpoIIIJ^C134A^-His_6_ purified from *E. coli* were loaded onto a Superose 12 HR 10/30 column (GE Healthcare) previously equilibrated with a buffer composed of 20 mM Tris-HCl pH 7.6, 10% glycerol, 0.5 M NaCl, 0.1% DDM, and 100 mM imidazole. The column was calibrated with the gel filtration molecular markers Dextran Blue, aldolase (158 kDa), ovalbumin (43 kDa), chymotryspinogen A (25 kDa), and lysozyme (14 kDa), in the same buffer used for equilibration. Fractions were subjected to immunoblot analysis.

### BN-PAGE

Blue-native polyacrylamide gel electrophoresis (BN-PAGE) was performed using gradient gels (5–15%) as described previously [33]. An elution fraction of 9 µg of His-tagged purified SpoIIIJ containing 100 mM imidazole and 500 mM NaCl was used. HMW-Native (Amersham Biosciences) was used as a molecular weight marker.

### Fluorescence microscopy

Samples (0.6 ml) of DSM cultures were collected at 4 and 6 h after the onset of sporulation, from cultures with (0.5 mM) or without IPTG, and resuspended in 0.2 ml of phosphate-buffered saline (8 mM sodium phosphate [pH 7.5], 150 mM NaCl) supplemented with 10 µg ml^−1^ of the lipophilic membrane dye FM4-64 (Molecular Probes) and with 0.2 µg ml^−1^ of the DNA dye 4-,6-diaminodino-2-phenylindole (DAPI). The cells were then observed by fluorescence microscopy [34], [35]. For the quantitative analysis of P*_sspE_-cfp* expression at least 200 cells were scored for the fluorescence patterns designated by low (class a) or high (class b). ImageJ (http://imagej.nih.gov/ij/) was used for quantification of the fluorescence signal. Low and high fluorescence refers to the intensity of the fluorescent signal as determined by a threshold defined with ImageJ, using σ^G^-inactive cells and σ^G^-active cells as references.

### MalPEG labelling of *B. subtilis* SpoIIIJ-His_6_

Cells grown in LB were collected by centrifugation, resuspended in 10 mM Tris-HCl pH 7.4, 1 mM EDTA, 1 mM PMSF, 1 mM Tris(2-carboxyethyl)phosphine (TCEP) and lysed with a French pressure cell (19 000 lb/in^2^). Membranes were isolated by a 60-min centrifugation at 100,000× *g*, resuspended in 50 mM Tris-HCl pH 6.8, 1 mM TCEP and further incubated either at 37°C or 80°C, with or without methoxypolyethylene glycol 5000 maleimide (malPEG), in the presence of 6 M urea and SDS (1 or 2%) or with no SDS nor urea. Other conditions for labelling of SpoIIIJ-His_6_ were as follows: 10% SDS; 2% SDS and 8 M Urea; 1% Triton and 4 M GdnHCl. Note that GdnHCl and SDS form a precipitate and could not be used in conjunction for this analysis.

### Whole-cell lysates and immunoblot analysis

*B. subtilis* strains were grown in DSM and samples collected one hour before the end of the growth phase (defined as the onset of sporulation, or T_0_), and two and four hours thereafter. Cells were resuspended in 10 mM Tris-HCl pH 7.4, 1 mM EDTA, 1 mM PMSF, 0.1 mM dithiothreitol (DTT) and lysed with a French pressure cell as above. Proteins (30 µg) were electrophoretically resolved on 12.5% polyacrylamide gels (SDS-PAGE) [36], transferred to nitrocellulose membranes and subjected to immunoblot analysis with a mouse anti-His_6_ antibody (Novagen, Darmstadt, Germany) for the detection of SpoIIIJ-His_6_ and YqjG-His_6_. The proteins were visualised with the ECL detection system (Amersham Biosciences) as described by the manufacturer.

### Topology prediction: SpoIIIJ's Cys134

TMPRED (http://www.ch.embnet.org/software/TMPRED_form.html) and TOP PRED (http://mobyle.pasteur.fr/cgibin/portal.py?form=toppred) were used to predict the localisation of SpoIIIJ Cys134 relative to the membrane plane (cytoplasmic/exterior/in the membrane).

## Results

### A role for Cys134 in the oligomerisation of SpoIIIJ

**Figure 1:**
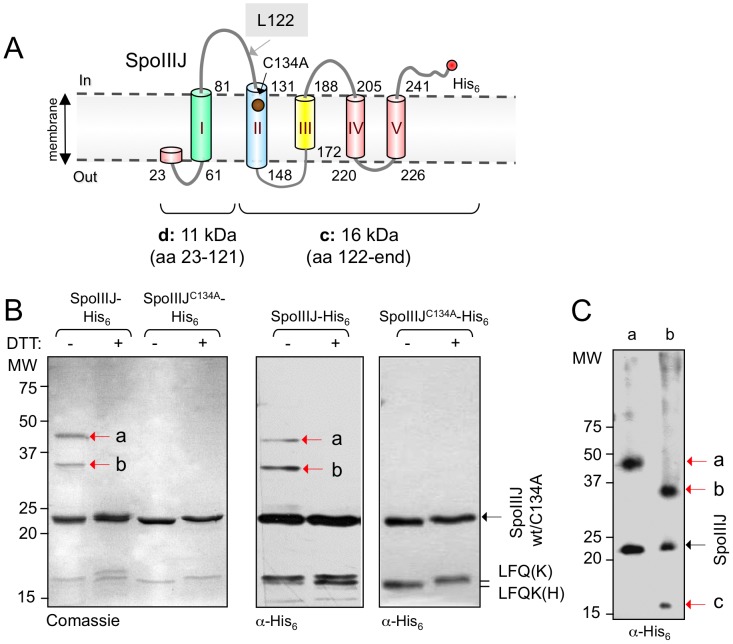
Dimerisation of SpoIIIJ-His_6_ purified from *E. coli*. (A) Topological model of SpoIIIJ in the membrane. The numbers refer to the amino acid residues that delimit the transmembrane (TM) segments, depicted as cylinders. Cys23, in the signal peptide is indicated, as well as Cys134 in TM2 (red circle). (B) SDS-PAGE and Coomassie-staining (left panel) or immunoblotting (right) with an anti-His_6_ antibody of purified SpoIIIJ-His_6_ and SpoIIIJ^C134A^-His_6_ in loading buffer with (100 mM) or without DTT. The N-terminal sequence of two processing products of SpoIIIJ-His_6_ is indicated. (C) Immunoblot analysis of SpoIIIJ-His_6_ species “a” and “b”, isolated from the gel in panel B (left), using an anti-His_6_ antibody. In panels B–C, the position of molecular weight markers (in kDa) is shown; black arrows show the position of full-length SpoIIIJ-His_6_ and red arrows the position of other SpoIIIJ forms.

We purified SpoIIIJ with a C-terminal His_6_ tag, SpoIIIJ-His_6_, from cells of *E. coli* C43(DE3). SpoIIIJ-His_6_ was solubilised with DDM from the membrane fraction obtained by ultracentrifugation and further purified over a Ni^2+^-column (Fig. S1A). About 0.8 mg/L of essentially pure SpoIIIJ-His_6_, as judged by SDS-PAGE analysis, was obtained (Fig. S1). SpoIIIJ is a polytopic membrane protein with 5 predicted transmembrane (TM) α-helices and a calculated molecular mass of 27 kDa (Fig. 1A). In agreement, analysis by far-UV circular dichroism indicates that the purified and detergent-solubilised SpoIIIJ-His_6_ corresponds to a folded α-helical rich protein (Fig. S2). Under reducing SDS-PAGE conditions, the most abundant form of the protein migrates with an apparent mass of 23 kDa (Fig. 1B) (see also [16]). However, higher molecular weight forms of SpoIIIJ-His_6_ were detected following Coomassie staining of SDS-PAGE gels, in the absence of the reducing agent DTT (Fig. 1B). Multimeric forms of SpoIIIJ-His_6_ have been observed before in the membrane fraction of *E. coli* cells over-producing this protein [24]. The high molecular weight forms of SpoIIIJ-His_6_ in Fig. 1B, have an apparent size of 30 and 45 kDa (Fig. 1B, bands “a” and “b”, respectively; these are indicated by red arrows). Immunoblotting with an anti-His_6_ antibody did not reveal additional forms (Fig. 1B; shorter forms of the protein are discussed below). This suggested that disulphide bonds between two cysteine residues of SpoIIIJ could be responsible for the formation of the 30 and 45 kDa forms.

In addition to the higher molecular weight forms of SpoIIIJ-His_6_ visualised in Fig. 1B (bands “a” and “b”), species migrating closely together at around 15 kDa were also seen, particularly by immunoblot analysis (Fig. 1B). The apparent molecular weight of band “a” is compatible with a dimer of full-length mature SpoIIIJ (that is, cleaved by signal peptidase II). Band “b” could, in turn, represent a heterodimer formed by full-length mature SpoIIIJ-His_6_ and one of the species found around the 15 kDa region of the gel. To test this hypothesis, bands “a” and “b” were excised from an SDS-PAGE gel and re-run separately. Starting with band “a”, both “a” and monomeric SpoIIIJ-His_6_ were detected by immunoblot analysis (Fig. 1C, red and black arrows, respectively). Thus, band “a” seems to be composed of two full-length mature SpoIIIJ monomers. Starting with band “b”, both “b” and monomeric SpoIIIJ-His_6_ were detected, along with this band, “c”, that seems to correspond to the slower migrating species of the doublet presumably resulting from processing of SpoIIIJ-His_6_ migrating just above the 15 kDa region of the gel (Fig. 1B and C). N-terminal sequencing of these two species of about 15 kDa, indicates that both start at residue L122 (Fig. 1B). These species are possibly generated by proteolytic cleavage of SpoIIIJ-His_6_ at L122, located in the cytoplasmic loop connecting TM1 and TM2, and removing TM1 (Fig. 1A). Cleavage at this position would generate fragments of 11 kDa (from Cys23 to part of the first cytoplasmic loop (including TM1), fragment “d” in Fig. 1A) and 16 kDa (the remainder of the first cytoplasmic loop to the end of the protein (including TM2), fragment “c” in Fig. 1A). The faster migrating species is not generated by a second cleavage event close to C-terminal of the larger species, as both are detected with an antibody against the C-terminally located His_6_ tag (Fig. 1B). Possibly, the difference in mobility of the doublet is caused by some other modification of the proteolytic product of SpoIIIJ-His_6_. In any event, these observations support the idea that SpoIIIJ-His_6_ purified from *E. coli* forms dimeric species. Moreover, they suggest that SpoIIIJ-His_6_ can form a heterodimer with a form of the protein lacking TM1.

SpoIIIJ-His_6_ has two Cys residues. One is located in the lipoprotein-type signal peptide and is modified during insertion of the protein into the membrane (Côrte and Henriques, unpublished data). A second, unmodified in mature SpoIIIJ-His_6_, is Cys134, located in TM2 (Fig. 1A). Cys134 in SpoIIIJ is conserved among orthologues of the protein, as shown in the alignment of Fig. S3A. A cysteine residue is found at the homologous position of YqjG (Cys142), the other member of the YidC/Oxa1/Alb3 family found in *B. subtilis*, and also in the *E. coli* YidC protein. As shown with several membrane topology prediction programs (see Materials and Methods), this residue seems to be always located within a TM segment (Fig. S3A). A helical wheel projection of the second TM segment of SpoIIIJ (Fig. S3B) showed it is a typical hydrophobic helix [37], [38]. This residue was the likely candidate for formation of disulphide bonds between SpoIIIJ monomers. To test this, we overproduced and purified a C-terminal His-tagged variant of SpoIIIJ in which Cys134 was substituted by an alanine (Fig. S1B). In contrast to the wild-type, no higher molecular weight bands of SpoIIIJ^C134A^-His_6_ were detected either by Coomassie-staining or immunoblotting of SDS-PAGE gels in the presence or absence of DTT (Fig. 1B). Far-UV CD analysis showed that the C134A substitution is not deleterious for the SpoIIIJ fold, as the spectrum obtained for this purified and detergent solubilised variant is essentially identical to that of the wild-type protein (Fig. S2A and B). Nevertheless, whereas reduction of SpoIIIJ-His_6_ with DTT yields minor secondary structure interconversions compatible with structural rearrangements upon reduction of the presumed C134-C134 disulphide, the same is not observed in the case of SpoIIIJ^C134A^-His_6_ (Fig. S2C).

**Figure 2:**
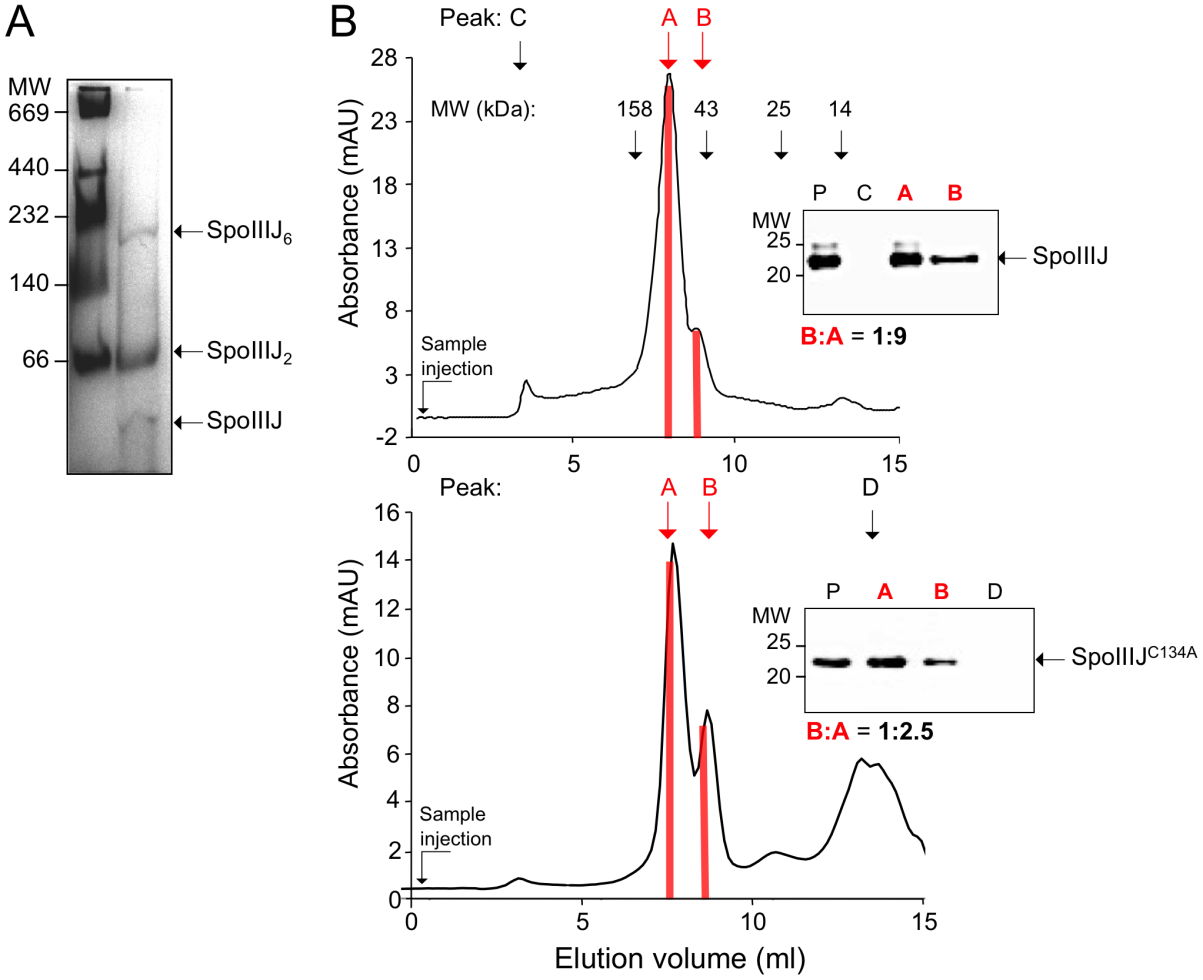
Oligomeric state of SpoIIIJ and SpoIIIJ^C134A^. (A) Blue-Native PAGE of purified SpoIIIJ-His_6_. Presumed hexameric (SpoIIIJ_6_), dimeric (SpoIIIJ_2_) and monomeric (SpoIIIJ) species are indicated. MW markers are shown in kDa. (B) Size exclusion chromatography of SpoIIIJ-His_6_ (top) or SpoIIIJ^C134A^-His_6_ (bottom) in the presence of 0.1% DDM and 500 mM NaCl at pH 7.6 (see the Material and Methods section for details). The vertical black arrows indicate the elution volumes of the size standards and additional peaks. The inserts show the immunoblot analysis of the peaks indicated in the two panels. Purified SpoIIIJ-His_6_ (top) or SpoIIIJ^C134A^-His_6_ (bottom) were included as a migration control (lane P). The areas of the A and B peaks in the two panels were estimated using the ImageJ software (http://imagej.nih.gov/ij/) and their ratio indicated.

As an independent test of oligomer formation by SpoIIIJ-His_6_, we used blue-native (BN) PAGE and size exclusion chromatography (SEC). By BN-PAGE, three species were observed that exhibited molecular weights compatible with a monomer, a dimer (the most represented species) and traces of a possible hexamer (Fig. 2A). SEC of purified SpoIIIJ-His_6_ revealed two main peaks (Fig. 2B) both of which contained the protein (as show by the immunoblot of the peak fractions; insert in Fig. 2B). The larger peak corresponds to a species with a calculated size (75 kDa) closer to that of a SpoIIIJ-His_6_ dimer (Fig. 2B). The smaller peak is caused by a species with a calculated size (47 kDa) closer to that of monomeric SpoIIIJ-His_6_ (Fig. 2B). The differences in these sizes from that estimated from BN-PAGE analysis probably results from the presence of dodecyl maltoside (DDM), the amount of which we were unable to estimate by SEC-MALLS (data not shown). Peaks with the same estimated sizes were also observed for SpoIIIJ^C134A^-His_6_ but in this case, the size of peak B, corresponding to the smaller species, increased relative to peak A (Fig. 2B). Quantification of the peak areas shows ratios of B to A of 1∶9 for SpoIIIJ-His_6_ and 1∶2.5 for SpoIIIJ^C134A^-His_6_.

Formation of the dimer size species (peak A) does not require Cys134 but taken all together our results suggest that Cys134 stabilises a multimeric (possibly a dimeric) form of SpoIIIJ-His_6_, in line with the suggestion that this residue is involved in formation of a disulphide bond between two monomers of the protein.

### Cys134 of SpoIIIJ is part of a protein-protein interface in *B. subtilis*

**Figure 3:**
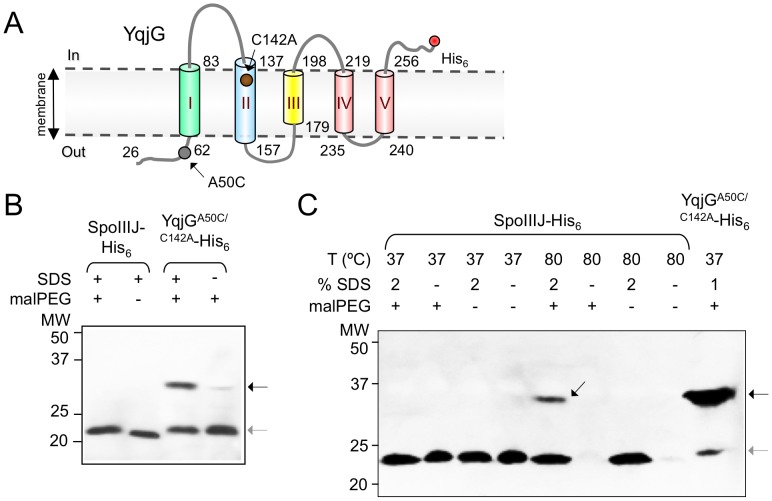
Labelling of YqjG^A50C/C142A^-His_6_ and SpoIIIJ-His_6_ with malPEG. (A) Topological model of YqjG in the membrane. The numbers refer to the amino acid residues that delimit the transmembrane (TM) segments I to V. The positions of the A50C and C142A mutations are indicated. (B) Strains producing SpoIIIJ-His_6_ or YqjG^A50C/C142A^-His_6_ in a *spoIIIJ* mutant background were grown in liquid LB, and samples withdrawn. Cells were resuspended in a buffer containing 1 mM TCEP, lysed and membranes isolated. The membranes were resuspended in the presence of 1 mM TCEP and further incubated with or without malPEG in the presence of 2% SDS (lanes 1–2), 1 or 0% SDS (lanes 3 and 4, respectively). Proteins (30 µg) were electrophoretically resolved by SDS-PAGE and immunoblotted with an anti-His_6_ antibody for the detection of SpoIIIJ-His_6_ and YqjG^A50C/C142A^-His_6_. (C) Samples from cultures of strains producing SpoIIIJ-His_6_ or YqjG^A50C/C142A^-His_6_ were withdrawn at the onset of the stationary growth phase in LB medium. Samples were treated as in (B), except that no TCEP was added to the French press buffer. Incubation proceeded at the indicated temperatures in the presence of 1 mM TCEP and with or without 1 mM malPEG and SDS, as indicated. In both B and C, grey and black arrows indicate SpoIIIJ-His_6_ or YqjG^A50C/C142A^-His_6_ or labeled (shifted) reaction products, respectively. The position of molecular weight markers (in kDa) is shown.

Cys134 is thought to be located in the plane of the membrane (Fig. 1A and S3), and involved in formation of a disulphide bond between SpoIIIJ-His_6_ monomers (Fig. 1). We wanted to examine whether Cys134 is located within the membrane and if it forms part of a protein-protein interaction interface. The membrane fraction of whole cell lysates prepared from *B. subtilis* cultures was isolated by ultracentrifugation and the available Cys residues in the proteins present in the sample were labelled with the Cys-modifying reagent methoxypolyethylene glycol 5000 maleimide (malPEG for simplicity). This reagent binds free sulfhydryl groups in cysteine residues forming thioether bonds, whilst adding an extra 5 kDa per available cysteine residue. As malPEG induces distortion in gel migration, the minimum concentration required to obtain appreciable labelling was determined and found to be 1 mM (data not shown). As malPEG is membrane-impermeable, SDS was also used to solubilise the membranes. If the cysteine is in the membrane plane, labelling was expected only in the presence of SDS. To increase the availability of free sulfhydryl groups by reducing eventual disulphide bonds, Tris(2-carboxyethyl)phosphine (TCEP) was used. Unlike DTT, this reagent does not compete with the cysteine residues for malPEG. As a positive control for labelling we used a substituted form of YqjG in which a Cys residue was introduced at position 50. As for the highly similar SpoIIIJ (Fig. 1A), this position is predicted to be located outside the cytoplasmic membrane, and facing the extracellular space (Fig. 3A). This residue should be more readily labelled with malPEG than a residue located in the plane of the membrane. Cys142 of YqjG (Fig. 3A) is homologous to Cys134 of SpoIIIJ and was substituted by an alanine to facilitate interpretation of the labelling pattern. Labelling of SpoIIJ with malPEG was not detected at 37°C even in the presence of 2% SDS (Fig. 3B) or at higher concentrations of the detergent (not shown). The level of SpoIIIJ in mixtures incubated at 80°C in the absence of SDS is strongly reduced (Fig. 3C, lanes 6 and 8), because under these conditions the protein failed to enter the gel (not shown). The combined action of detergents and denaturants has been shown to disclose normally hidden residues [39], [40]. However, none of the combinations herein tested (GdnHCl, urea, Triton and SDS), result in labelling of Cys134 of SpoIIIJ-His_6_ (see Materials and Methods). However, SpoIIIJ-His_6_ was labelled at 80°C in the presence of 2% SDS (Fig. 3C). Thus, high temperature and the presence of a detergent are required for labelling Cys134 of SpoIIIJ-His_6_ with malPEG. In contrast, labelling of the positive control YqjG^A50C/C142A^-His_6_ was obtained in the presence of 1% SDS and also in its absence (Fig. 3B and C). We observed a shift in the apparent molecular weight for malPEG-derivatised YqjG^A50C/C142A^-His_6_ larger than the expected 5 kDa, which has also been reported previously [41].

Together, these results are compatible with the view that Cys134 is located within the membrane plane, and is part of a tight protein-protein interaction surface.

### Accumulation of wild-type and substituted SpoIIIJ during sporulation in *B. subtilis*

**Figure 4:**
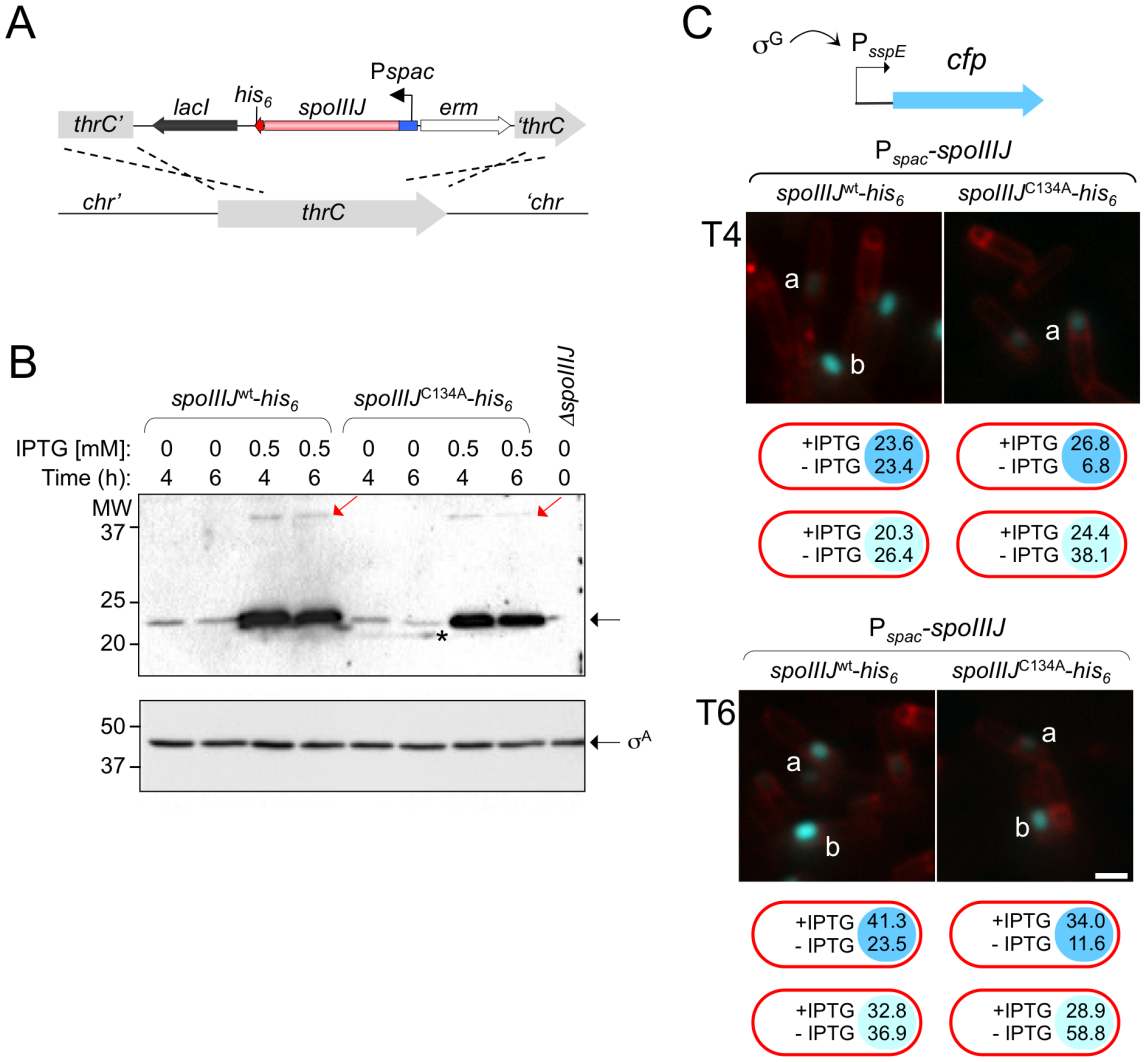
Effect of *spoIIIJ* ^C134A^ on sporulation. (A) Schematic representation of the double crossover integration of a P*_spac_-spoIIIJ-his_6_* fusion at the non-essential *thrC* locus. (B) Strains expressing SpoIIIJ-His_6_ or SpoIIIJ^C134A^-His_6_ from P*_spac_* at *thrC* (as shown in A) in a Δ*spoIIIJ* background, were grown in parallel with a Δ*spoIIIJ* mutant (last lane) in liquid DSM with or without IPTG as indicated and samples withdrawn at hours 4 and 6 of sporulation. Proteins in whole-cell extracts were subject to immunoblot analysis with an anti-His_6_ (upper panel) or an anti-σ^A^ antibody (lower panel). The samples were resolved by SDS-PAGE under reducing conditions. The position of SpoIIIJ-His_6_, SpoIIIJ^C134A^-His_6_ or σ^A^ is indicated by black arrows. The red arrows show the position of presumed SpoIIIJ-His_6_ or SpoIIIJ^C134A^-His_6_ dimers. The asterisk indicates a non-specific cross-reacting species. The position of molecular weight markers (in kDa) is shown. (C) Expression of a P*_sspE_*-*cfp* fusion, used as a reporter for σ^G^ activity, in DSM cultures of strains expressing wild-type *spoIIIJ* or the *spoIIIJ* ^C134A^ allele from the P*_spac_* promoter at *thrC* in a Δ*spoIIIJ* background. The strains were grown in the absence or in the presence of IPTG (0.5 mM). Only images for the non-induced strains are shown. Samples were collected at the indicated times (in hours) after the onset of sporulation, stained with the membrane dye FM4-64 and observed by fluorescence microscopy. CFP and FM4-64 fluorescence are shown in blue and red, respectively. The fluorescence patterns were designated by low (class *a*) or high (class *b*) fluorescence. Scale bar, 1 µm. The cartoons below each panel show the percentage of cells in class *a* (light blue) or class *b* (darker blue) for each of the indicated strains grown in the absence (“−”) or in the presence (“+”) of 0.5 mM IPTG, as indicated. At least 200 cells were scored for each of the *a* or *b* classes (see the Material and Methods section).

*spoIIIJ* is required for sporulation [10]. To evaluate the ability of SpoIIIJ^C134A^ to support efficient sporulation, both the wild-type and C134A form of SpoIIIJ were expressed from the IPTG-inducible P*_spac_* promoter, placed at the non-essential *thrC* locus in a *spoIIIJ* deletion background (Fig. 4A). When measured 24 hours after the onset of sporulation in DSM, the non-induced C134A strain produced very low levels of heat-resistant spores when compared to its wild-type counterpart (Table 1). Importantly, the level of heat-resistant spores for the non-induced wild-type allele was close to that found for a wild-type strain (*i.e*., expressing *spoIIIJ* from its normal locus) (Table 1). In contrast, upon addition of IPTG both the wild-type and the C134A mutant exhibited sporulation levels similar to a wild-type strain (Table 1). The fact that a low spore titre was observed for the non-induced mutant but not for the non-induced wild-type protein, suggested that under these conditions SpoIIIJ^C134A^-His_6_ accumulates to lower levels or is less functional than the wild-type form. To distinguish between these possibilities, we analysed the levels of either protein under inducing and non-inducing conditions. When induced, both the wild-type and C134A forms of SpoIIIJ accumulate to levels close to those of the protein produced from its native locus (Fig. S4). When non-induced, the levels of SpoIIIJ or SpoIIIJ^C134A^ are both barely detectable, but similar to one another (Fig. 4B). Hence, it seems unlikely that different expression levels account for the difference in spore titre observed for the two forms of the protein (Table 1). Note that a species with the expected size for a dimer is seen for both SpoIIIJ^wt^-His_6_ and SpoIIIJ^C134A^-His_6_ (Fig. 4B, red arrows). The representation of this species did not increase when the experiment was repeated in the absence of DTT, suggesting that Cys134 is not sensitive to the reducing agent (data not shown), in agreement with the idea that this residue is part of a tight protein-protein interaction surface (Fig. 3; above). Because the level of heat-resistant spores for the non-induced wild-type allele was close to that found for a wild-type strain (Table 1, see also above), the data shows that low levels of wild-type SpoIIIJ are sufficient for efficient sporulation. Together, these results suggest that SpoIIIJ^C134A^ is less functional than the wild-type protein, and that this reduced functionality emerges at low expression levels of the corresponding alleles.

**Table 1:** Efficiency of sporulation^a^ of strains bearing various *spoIIIJ* alleles.

Strain	Relevant Genotype	[IPTG] mM	Viable cell count^b^	Heat^R^ cell count	Spo (%)
MB24	wild-type	0	3.4×10^8^	2.3×10^8^	68.3
			±2.3×10^8^	±3.0×10^8^	
AH5425	*spoIIIJ* Δ*spoIIIJ*::*km*	0	5.6×10^7^	1.3×10^7^	23.7
			±7.1×10^6^	±3.8×10^6^	
AH5426	*spoIIIJ* ^C134A^ Δ*spoIIIJ*::*km*	0	4.6×10^7^	1.2×10^6^	2.5
			±8.7×10^6^	±1.8×10^5^	
AH5425	*spoIIIJ* Δ*spoIIIJ*::*km*	0.5	±1.8×10^8^	1.2×10^8^	68
			±1.5×10^7^	±1.7×10^7^	
AH5426	*spoIIIJ* ^C134A^ Δ*spoIIIJ*::*km*	0.5	7.9×10^7^	3.5×10^7^	43.5
			±1.4×10^7^	±8.1×10^6^	
JOB44	Δ*spoIIIJ*::*km*	0	4.8×10^7^	0	0
			±7.6×10^6^		

a The titre of viable cells and heat-resistant spores was measured 24 h after the onset of sporulation in DSM (see Materials and Methods).

b The values are averages ± SD, for three independent experiments.

### Requirement of Cys134 of SpoIIIJ for σ^G^ activity

Since SpoIIIJ is required for late forespore-specific gene expression during sporulation [10], we wanted to assess the impact of the C134A substitution on the activity of σ^G^. We used the σ^G^-controlled transcriptional fusion P*_sspE_*-*cfp* [23] to monitor the activity of σ^G^ in single cells by fluorescence microscopy (Fig. 4C). DSM cultures of strains expressing either *spoIIIJ* or *spoIIIJ* ^C134A^ from P*_spac_* in a *spoIIIJ* mutant background were sampled at hours 4 and 6 after the onset of sporulation. The cells were observed by fluorescence microscopy after staining with the membrane dye FM4-64 and the nucleoid stain DAPI (see Materials and Methods), to identify and score the morphological stage of sporulation. Sporulating cells were assigned to two classes according to the intensity of the CFP signal: with a low (*a*) and with a high (*b*) fluorescence signal (Fig. 4C). High-fluorescent cells completed engulfment and activated σ^G^, thus leading to high expression of the reporter fusion P*_sspE_*-*cfp*; on the other hand, low fluorescence mainly results from the weaker utilization of the *sspE* promoter by the preceding sigma factor, σ^F^, in the forespore line of gene expression [24].

For the non-induced strain bearing the P*_spac_*-*spoIIIJ* allele 23.4% of the cells at hour 4 of sporulation, and 23.5% of the cells at hour 6 were scored as highly fluorescent (class *b*), an indication of active σ^G^; in contrast, the P*_spac_-spoIIIJ* ^C134A^-expressing strain under non-inducing conditions showed a large proportion of low-fluorescence cells (class *a*; 38.1% at hour 4 and 58.8% at hour 6), whilst the high-fluorescence cells were scarce (class *b*; 6.8% at hour 4 and 11.6% at hour 6) (Fig. 4C). A distribution similar to that of the non-induced P*_spac_-spoIIIJ* ^C134A^ strain was obtained for a *spoIIIJ* null mutant (not shown), as expected, as in these cells only σ^F^ is active [10]. Upon addition of IPTG (to 0.5 mM), similar percentages of highly fluorescent, class *b* cells were obtained for the strains expressing either *spoIIIJ* ^C134A^ (26.8% at hour 4 and 34% at hour 6) or its wild-type counterpart (23.6% and 41.3% at hour 6, respectively) (Fig. 4C). Together, the results show that expression of *spoIIIJ* ^C134A^ does not cause a delay in sporulation. Rather, the results suggest that the C134A substituted form of SpoIIIJ is less active than the wild-type protein, but the lower activity of *spoIIIJ* ^C134A^ is evident *in vivo* only when lower than normal levels of the proteins is produced.

## Discussion

**Figure 5:**
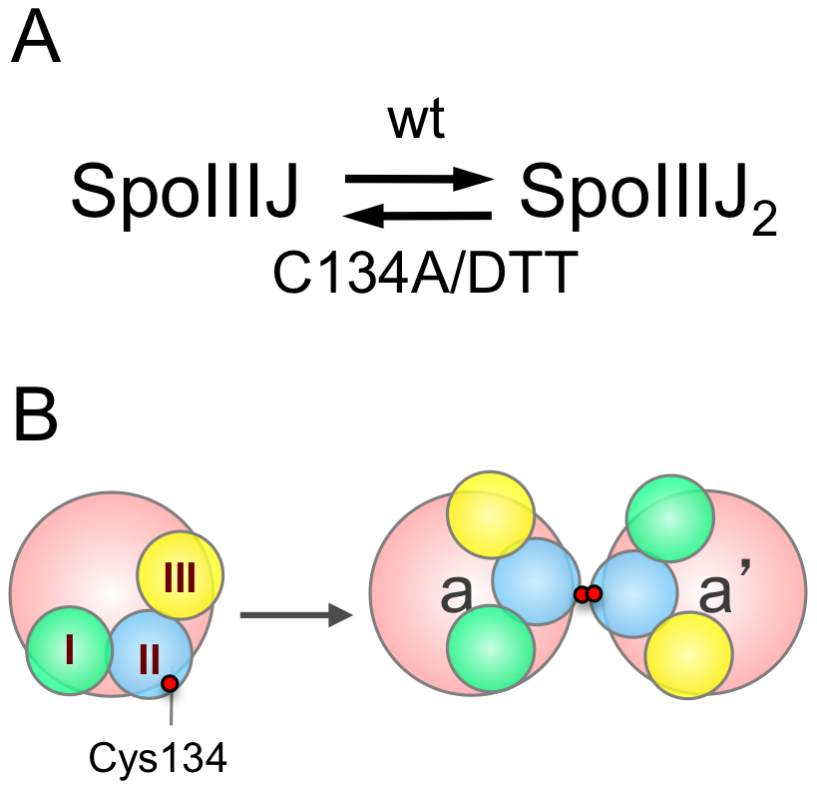
Dimerisation of SpoIIIJ. (A) SpoIIIJ occurs as a monomer and a dimer. The dimer is thought to be stabilised by a disulphide bond involving residue Cys134. However, this residue is not essential for dimer formation. (B) A disulphide bond between cysteine residues (red circles), along with other interactions promotes the formation and/or maintenance of the dimeric form of SpoIIIJ. TM segments 1, 2 and 3 are depicted as green, blue, and yellow circles, respectively.

*B. subtilis* SpoIIIJ, a member of the YidC/Oxa1/Alb3 family of insertases, forms a dimer. The Cys134 residue, predicted to reside within the TM2 segment of SpoIIIJ, has a role in dimer formation, presumably by establishing a disulphide bond. Several lines of evidence suggest that formation of an intramembrane disulphide bond, involving Cys134 promotes formation of, or stabilises a SpoIIIJ dimer. SpoIIIJ was detected as a monomer and a dimer by SDS-PAGE, but only a monomeric species was seen in the presence of the reducing agent DTT (Fig. 5A). In contrast, SpoIIIJ^C134A^, in which the only Cys residue in mature SpoIIIJ was changed to an Ala, migrated as a monomeric species, even in the absence of DTT (Fig. 5A). Also, the monomer∶dimer ratio, as estimated by SEC, was higher for SpoIIIJ^C134A^ in comparison to the wild-type protein. Circular dichroism spectroscopy analysis suggested that DDM-solubilised SpoIIIJ is a folded, mainly α-helical protein, and we found that the addition of DTT caused an alteration in the content of α-helices and anti-parallel β-sheets for SpoIIIJ but not for SpoIIIJ^C134A^. This change in secondary structure may be explained, at least in part, by a change in the monomer∶dimer ratio. Clearly, despite its importance, Cys134 is not absolutely required for dimer formation. Rather, the dimer appears to be sustained by a disulphide bond along with other non-covalent interactions. Oligomerisation is a widespread mechanism for regulating protein function [42]. For SpoIIIJ, dimer formation is important, at least for sporulation (see also below), and an inter-chain disulphide bond may be advantageous as it might stabilise the functional form of the protein. More generally, inter-chain disulphide bonds may help coping with naturally occurring mutations that reduce the potential for dimerisation or oligomerisation [43]. The Cys134 residue is predicted to occur within the membrane plane, in TM2. Conservation of a Cys residue at a homologous position in the YqjG paralogue, and among SpoIIIJ orthologues, underscores the importance of this residue, and presumably of disulphide bond formation for optimal functionality.

It is often assumed that the membrane environment does not favour formation of disulphide bonds, but an increasing number of reports argue in favour of the existence of such intramembrane disulphide bonds [*e.g.*], [ 44], [45], [46,47]. The experiments using malPEG were designed to test whether a free cysteine residue was accessible for modification in SpoIIIJ. We have found that labelling with malPEG only took place by exposing the protein to high temperature in the presence of high concentrations of SDS in (Fig. 3). The harsh conditions required for labelling of Cys134 suggest that this residue is present at a tight interface, as the bulky malPEG molecule may have only limited access to the cysteine residue [39]. This may contribute to the incomplete labelling observed (Fig. 3). Since the labelling experiments were implemented with extracts prepared from *B. subtilis* cultures, another factor contributing for incomplete labelling could be the existence of a mixed population of SpoIIIJ in several oligomeric states and/or belonging to distinct protein complexes allowing different degrees of malPEG accessibility.

Distinct oligomeric states have been reported for members of the YidC/Oxa1/Alb3 family of membrane protein insertases. Oxa1 from *Neurospora crassa* was suggested to form tetramers [48] and Oxa1 from *S. cerevisiae* was observed to form only dimers [9], only tetramers [49], or dimers and tetramers [50]. Alb3 from *Arabidopsis thaliana* is able to form dimers [51]. Finally, YidC from *E. coli* was reported to form dimers [9], [52], [53], [54] and was also purified as a mixture of monomers and dimers [55], [56]. We cannot exclude that forms of SpoIIIJ of order higher than the dimer are formed and have physiological relevance. The BN-PAGE, for examples, suggests that a SpoIIIJ hexamer can be formed, and at least part of the dimer population could serve as an intermediate for the formation of this species. However, we cannot presently exclude that the hexamer is only formed due to overproduction of the protein in *E. coli*.

A role for TM1 of SpoIIIJ (corresponding to TM2 of YidC) in SpoIIIJ-SpoIIIJ interactions is hinted from the fact that a heterodimer composed of a full-length, mature, and a truncated SpoIIIJ monomer (lacking TM1) was observed but not a homodimer of two truncated SpoIIIJ monomers (Fig. 1). This role for TM1 may be direct or indirect. TM1 could itself be involved in protein-protein interactions, or it could maintain the regions directly interacting (possibly TM2, with C134) in the correct position (Fig. 5B). Interestingly, TM2 of YidC (corresponding to SpoIIIJ's TM1) was suggested to have a structural role as well, in helix-helix interactions [57]. In addition, an interaction between TM2 and 3 of YidC (corresponding to TM1 and 2 of SpoIIIJ, respectively) was also suggested [58]. Importantly, a recent study by Geng and co-authors in which a series of SpoIIIJ/YqjG chimeras were analysed [59], also highlights the critical role of TM2 of SpoIIIJ (containing Cys134) for its role during sporulation.

Both SpoIIIJ and YidC function together with or independently of the Sec system [reviewed in 3], [60]. For instance, SpoIIIJ participates in the biogenesis of the SpoIIIAE polytopic membrane protein, one of the components of the secretion system required for normal σ^G^ activity after engulfment completion, possibly in conjunction with the Sec pathway [20], [24]. Depending on either mode of action, in conjunction with or independently of the Sec pathway, SpoIIIJ may function mostly as a monomer or mostly as a dimer. The precise role fulfilled by SpoIIIJ during membrane protein biogenesis could also vary regarding different substrates, either in guiding membrane protein insertion or later, in the folding or the assembly of membrane protein complexes [6]. Our observation that at low expression levels, SpoIIIJ^C134A^ but not the wild-type protein, shows a reduced ability to support σ^G^ activity and sporulation (Fig. 4 and Table 1), may suggest that a higher concentration of the mutant protein is needed to promote dimer formation, as the disulphide bond cannot be formed. Dimeric (or oligomeric) SpoIIIJ may be the form of the protein that best functions in the assembly of the SpoIIIA-SpoIIQ-based secretion system.

The basis for the observation that SpoIIIJ but not its paralogue YqjG supports efficient sporulation is not fully understood. Since a positional homologue of Cys134 is found in YqjG, it seems possible that this residue is also involved in disulphide bond formation in this protein. If so, formation of a disulphide bond between proximal TM2 segments does not distinguish SpoIIIJ and YqjG with respect to sporulation. Other regions of SpoIIIJ may be important, as a class of suppressor mutations that permit σ^G^ activation and sporulation in the absence of *spoIIIJ* map to TM5 and to the loop between TM4 and TM5 of *yqjG* [20].

## Supporting Information

Figure S1 **Overproduction and purification of SpoIIIJ-His_6_ and SpoIIIJ^C134A^-His_6_.** Overproduction and purification of SpoIIIJ-His_6_ (A) or SpoIIIJ^C134A^-His_6_ (B) from *E. coli* strain C43(DE3) carrying pMS266 or pFV1, respectively. The cells were grown in LB to mid log phase, split into two cultures, and one was induced with IPTG. The cells were lysed and fractionated into a soluble and a membrane fraction. Proteins in the membrane fraction were solubilised with 2% DDM and the extract applied onto a Ni^2+^-NTA column. (A) The fractions analysed by SDS-PAGE are as follows: lanes 1 and 2, crude extract of non-induced and induced cells, respectively; lanes 3 and 4, membrane fraction extracted with 2% DDM from non-induced and induced cells, respectively; lane 5, column flow through; lane 6, column wash; lane 7, 50 mM imidazole elution fraction; lane 8, 100 mM imidazole fraction. (B) lanes 1 and 2, crude extract of non-induced and induced cells, respectively; lane 3, membrane extract solubilised with 2% DDM; lane 4, flow through; lane 5, column wash; lane 6, 25 mM imidazole elution fraction; lanes 7 and 8, 50 mM imidazole elution. The position of molecular weight markers (in kDa) is shown; arrows show the position of full-length SpoIIIJ-His_6_ or SpoIIIJ^C134A^-His_6_.
(TIF)

Figure S2 **Circular dichroism spectroscopy of SpoIIIJ.** Far UV-CD spectra of purified SpoIIIJ-His_6_ (A) or SpoIIIJ^C134A^-His_6_ (B) (0.2 mg/ml of purified protein in 20 mM Tris-HCl pH 8, 0.1 M NaCl, 10% glycerol) in the presence or the absence of 1 mM DTT, dotted and solid lines, respectively. The spectra are typical of folded α-helical rich proteins with minima at 208 and 222 nm, consistent with the predicted structure of SpoIIIJ. Addition of DTT affects the spectrum of SpoIIIJ-His_6_, but has no effect on the spectrum of SpoIIIJ^C134A^-His_6_. (C) Relative estimates of the secondary structure of SpoIIIJ-His_6_ (with or without 1 mM DTT, dark grey and black bars, respectively) and SpoIIIJ^C134A^-His_6_ (with or without 1 mM DTT, white and light grey bars, respectively). Addition of DTT decreases the α-helix content by 6% and increases the content of antiparallel β-sheets (more 8%) of SpoIIIJ-His_6_, but does not significantly alter SpoIIIJ^C134A^-His_6_.
(TIF)

Figure S3 **Conservation of Cys134 among SpoIIIJ orthologues.** (A) Alignment of the transmembrane (TM) segment 2 of SpoIIIJ (red) and YqjG (green) proteins from several *Bacillus* species and of TM3 of YidC (blue) from *E. coli*. Conserved residues are shaded in grey except for the cysteine (yellow). The sequences were aligned with ClustalW [61]. (B) Helical wheel projection of TM2 of SpoIIIJ from *B. subtilis* (http://rzlab.ucr.edu/scripts/wheel/wheel.cgi). Circles denote hydrophilic residues and diamonds hydrophobic ones. The color code is as follows: green, hydrophobic residues, with the amount of green decreasing proportionally to the hydrophobicity; yellow, zero hydrophobicity; red, the most hydrophilic (uncharged) residue, the amount of red decreasing proportionally to the hydrophilicity.
(TIF)

Figure S4 **Expression of SpoIIIJ-His_6_ and SpoIIIJ^C134A^-His_6_ in** ***B. subtilis*** **.** The figure compares the levels of SpoIIIJ-His_6_ and SpoIIIJ^C134A^-His_6_ expressed from the *thrC* locus under the control of P*_spac_*, with the level of SpoIIIJ-His_6_ expressed from the *spoIIIJ* locus under the control of its normal promoter (identified by the “*” symbol). The cultures were grown in liquid DSM in the presence (for the fusions at *thrC*) or in the absence of IPTG (for the fusion at the *spoIIIJ* locus), and samples withdrawn at hours 4 and 6 of sporulation. Proteins in whole-cell extracts were subject to immunoblot analysis with an anti-His_6_ (upper panel) or an anti-σ^A^ antibody (lower panel). The gels were run under reducing conditions. The positions of SpoIIIJ-His_6_ (wt or C134A) and σ^A^ are indicated by black arrows; red arrows show the position of presumed SpoIIIJ dimers. The position of molecular weight markers (in kDa) is shown. Note that the SpoIIIJ-His_6_* fusion, expressed from the *spoIIIJ* locus under the control of its normal promoter, has a linker longer than the fusions expressed under the control of P*_spac_* at *thrC* locus, and shows a slightly higher apparent mass [24].
(TIF)

Table S1 **Bacterial strains.**
(DOC)

Table S2 **Oligonucleotides used in this study.**
(DOC)

Table S3 **Plasmids.**
(DOC)
