# Supplementary figures and images for: A Conserved Cysteine Residue of Bacillus subtilis SpoIIIJ Is Important for Endospore Development
Source: PLoS One. 2014 Aug 18;9(8):e99811. doi: 10.1371/journal.pone.0099811 (PMC4136701; doi:10.1371/journal.pone.0099811)

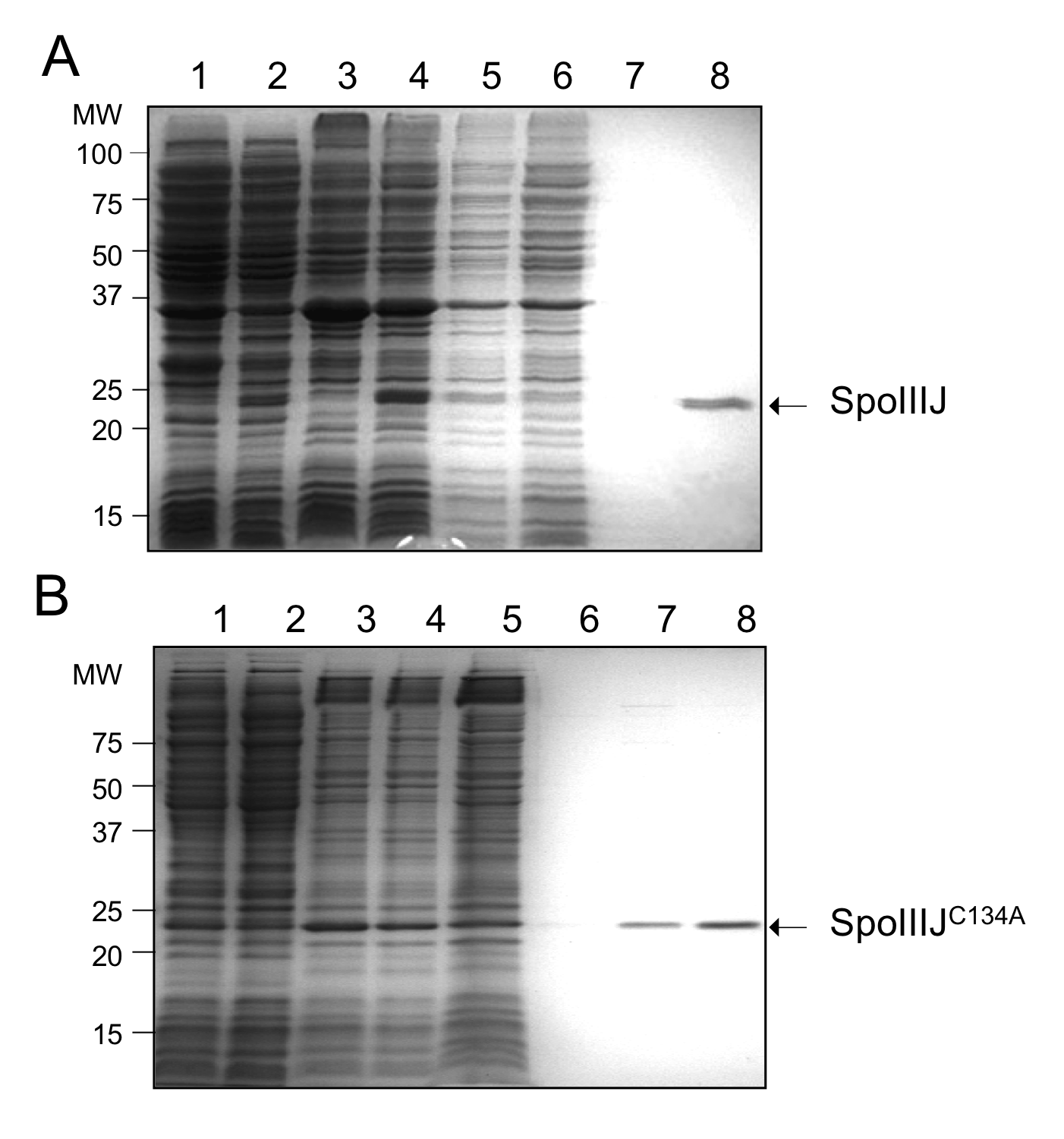

Supplement: Figure S1 — Overproduction and purification of SpoIIIJ-His6 and SpoIIIJC134A-His6. Overproduction and purification of SpoIIIJ-His6 (A) or SpoIIIJC134A-His6 (B) from E. coli strain C43(DE3) carrying pMS266 or pFV1, respectively. The cells were grown in LB to mid log phase, split into two cultures, and one was induced with IPTG. The cells were lysed and fractionated into a soluble and a membrane fraction. Proteins in the membrane fraction were solubilised with 2% DDM and the extract applied onto a Ni2+-NTA column. (A) The fractions analysed by SDS-PAGE are as follows: lanes 1 and 2, crude extract of non-induced and induced cells, respectively; lanes 3 and 4, membrane fraction extracted with 2% DDM from non-induced and induced cells, respectively; lane 5, column flow through; lane 6, column wash; lane 7, 50 mM imidazole elution fraction; lane 8, 100 mM imidazole fraction. (B) lanes 1 and 2, crude extract of non-induced and induced cells, respectively; lane 3, membrane extract solubilised with 2% DDM; lane 4, flow through; lane 5, column wash; lane 6, 25 mM imidazole elution fraction; lanes 7 and 8, 50 mM imidazole elution. The position of molecular weight markers (in kDa) is shown; arrows show the position of full-length SpoIIIJ-His6 or SpoIIIJC134A-His6. (TIF) [file pone.0099811.s001.tif]

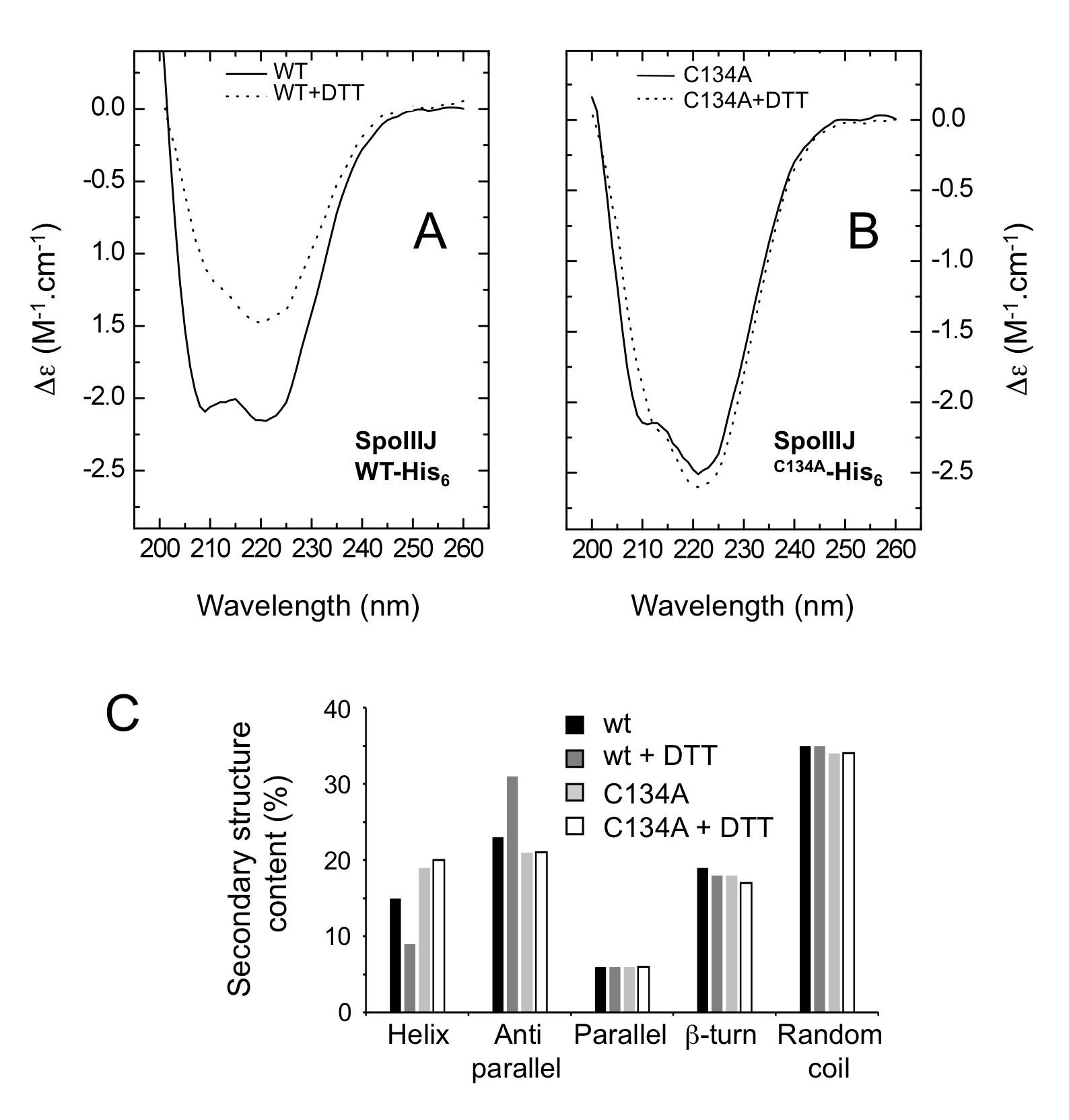

Supplement: Figure S2 — Circular dichroism spectroscopy of SpoIIIJ. Far UV-CD spectra of purified SpoIIIJ-His6 (A) or SpoIIIJC134A-His6 (B) (0.2 mg/ml of purified protein in 20 mM Tris-HCl pH 8, 0.1 M NaCl, 10% glycerol) in the presence or the absence of 1 mM DTT, dotted and solid lines, respectively. The spectra are typical of folded α-helical rich proteins with minima at 208 and 222 nm, consistent with the predicted structure of SpoIIIJ. Addition of DTT affects the spectrum of SpoIIIJ-His6, but has no effect on the spectrum of SpoIIIJC134A-His6. (C) Relative estimates of the secondary structure of SpoIIIJ-His6 (with or without 1 mM DTT, dark grey and black bars, respectively) and SpoIIIJC134A-His6 (with or without 1 mM DTT, white and light grey bars, respectively). Addition of DTT decreases the α-helix content by 6% and increases the content of antiparallel β-sheets (more 8%) of SpoIIIJ-His6, but does not significantly alter SpoIIIJC134A-His6. (TIF) [file pone.0099811.s002.tif]

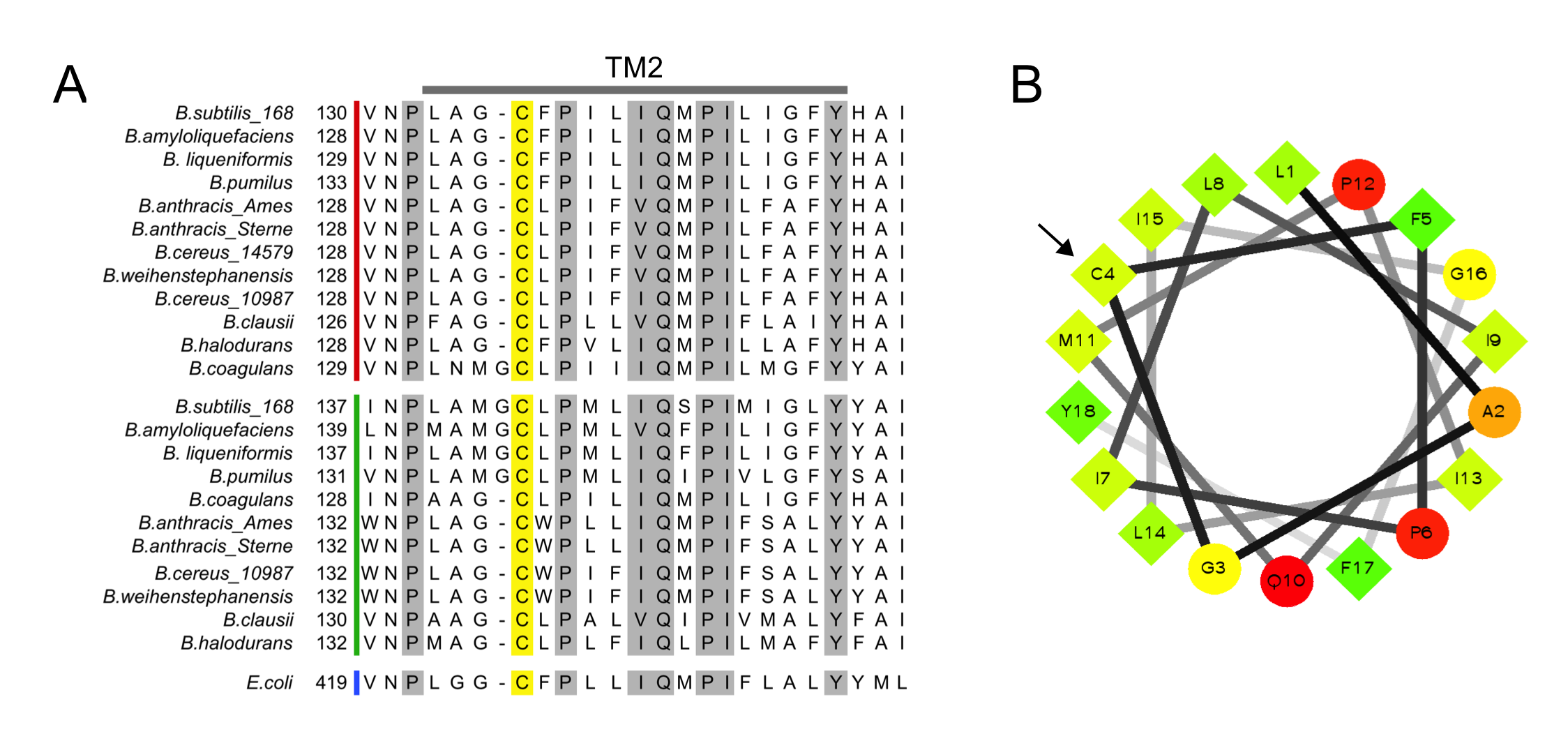

Supplement: Figure S3 — Conservation of Cys134 among SpoIIIJ orthologues. (A) Alignment of the transmembrane (TM) segment 2 of SpoIIIJ (red) and YqjG (green) proteins from several Bacillus species and of TM3 of YidC (blue) from E. coli. Conserved residues are shaded in grey except for the cysteine (yellow). The sequences were aligned with ClustalW [61]. (B) Helical wheel projection of TM2 of SpoIIIJ from B. subtilis (http://rzlab.ucr.edu/scripts/wheel/wheel.cgi). Circles denote hydrophilic residues and diamonds hydrophobic ones. The color code is as follows: green, hydrophobic residues, with the amount of green decreasing proportionally to the hydrophobicity; yellow, zero hydrophobicity; red, the most hydrophilic (uncharged) residue, the amount of red decreasing proportionally to the hydrophilicity. (TIF) [file pone.0099811.s003.tif]

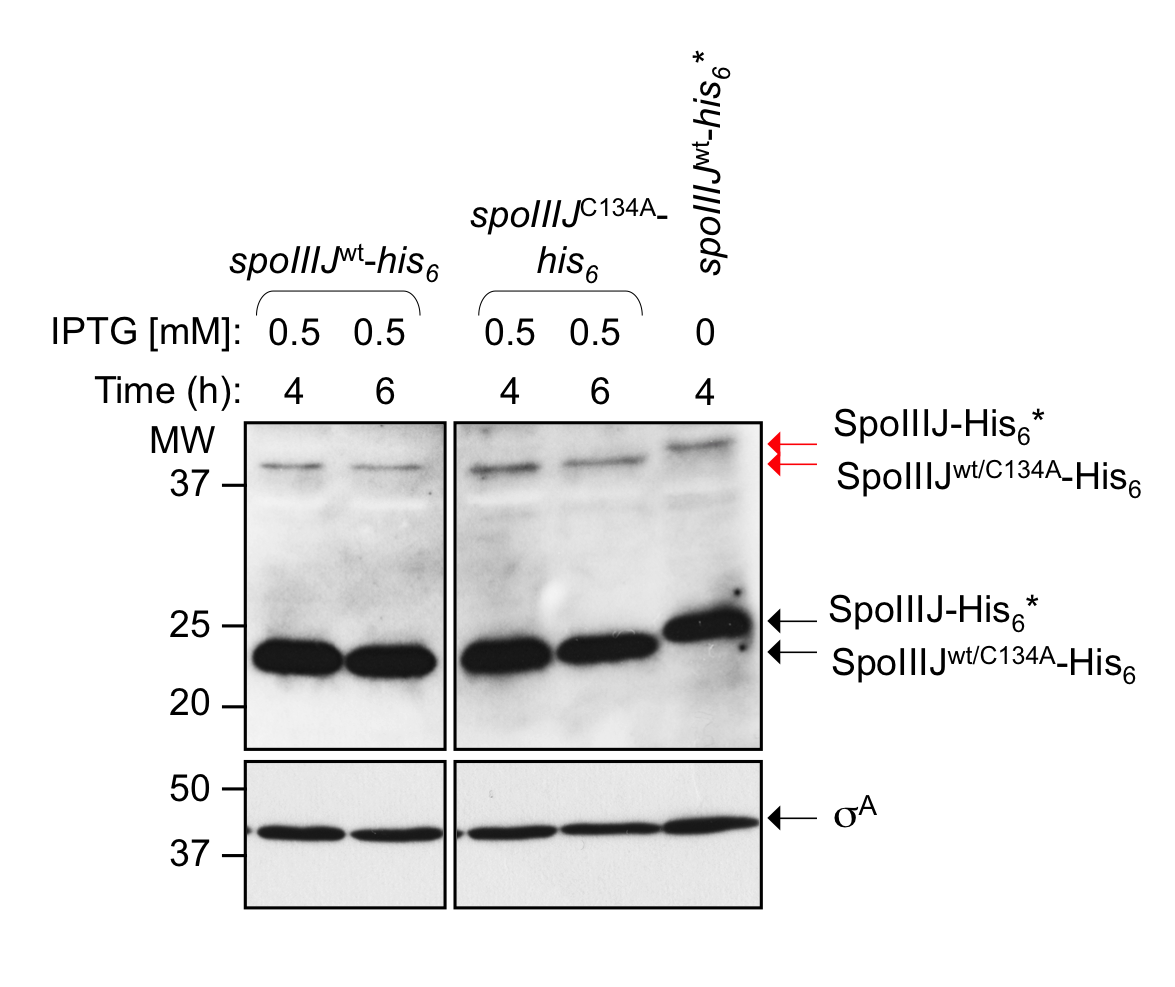

Supplement: Figure S4 — Expression of SpoIIIJ-His6 and SpoIIIJC134A-His6 in B. subtilis . The figure compares the levels of SpoIIIJ-His6 and SpoIIIJC134A-His6 expressed from the thrC locus under the control of Pspac, with the level of SpoIIIJ-His6 expressed from the spoIIIJ locus under the control of its normal promoter (identified by the “*” symbol). The cultures were grown in liquid DSM in the presence (for the fusions at thrC) or in the absence of IPTG (for the fusion at the spoIIIJ locus), and samples withdrawn at hours 4 and 6 of sporulation. Proteins in whole-cell extracts were subject to immunoblot analysis with an anti-His6 (upper panel) or an anti-σA antibody (lower panel). The gels were run under reducing conditions. The positions of SpoIIIJ-His6 (wt or C134A) and σA are indicated by black arrows; red arrows show the position of presumed SpoIIIJ dimers. The position of molecular weight markers (in kDa) is shown. Note that the SpoIIIJ-His6* fusion, expressed from the spoIIIJ locus under the control of its normal promoter, has a linker longer than the fusions expressed under the control of Pspac at thrC locus, and shows a slightly higher apparent mass [24]. (TIF) [file pone.0099811.s004.tif]
