# Supplementary material for: A Conserved Cysteine Residue of Bacillus subtilis SpoIIIJ Is Important for Endospore Development
Source: PLoS One. 2014 Aug 18;9(8):e99811. doi: 10.1371/journal.pone.0099811 (PMC4136701; doi:10.1371/journal.pone.0099811)
Supplement: Table S1 — Bacterial strains. (DOC) [file pone.0099811.s005.doc]

**A conserved cysteine residue of *Bacillus subtilis* SpoIIIJ is important for endospore development**

Luísa Côrte**^1^**, Filipa Valente**^1*^**, Mónica Serrano**^1^**, Cláudio M. Gomes**^1^**, Charles P. Moran, Jr**^3^**., and Adriano O. Henriques**^1,3^**

Instituto de Tecnologia Química e Biológica, Universidade Nova de Lisboa,

Avenida da República, Apartado 127, 2oz781-901 Oeiras, Portugal**^1^**, and Department of Microbiology and Immunology, Emory University School of Medicine, Atlanta, Georgia 303222**^2^**

**Supporting information – Table S1**

**Table S**1. Bacterial strains.

| **Strain** | Relevant Genotype/Phenotype^a^ | Origin/Reference |
| --- | --- | --- |
| MB24 | *trpC2 metC3* | Laboratory stock |
| JOB44 | *trpC2 metC3* Δ*spoIIIJ*::*km* / Km^r^ | [17] |
| AH5382 | *trpC2 metC3* Δ*spoIIIJ*::*km* Δ*amyE*::*yqjG*^A50C/C142A^-*his_6_* / Km^r^  Cm^r^ | This work |
| AH5425 | *trpC2 metC3* Δ*spoIIIJ*::*km* Δ*thrC*::P*_spac_spoIIIJ*-*his_6_* / Km^r^  Erm^r^ | « |
| AH5426 | *trpC2 metC3* Δ*spoIIIJ*::*km* Δ*thrC*::P*_spac_spoIIIJ*^C134A^-*his_6_* / Km^r^  Erm^r^ | « |
| AH5431 | *trpC2 metC3* Δ*spoIIIJ*::*km* Δ*thrC*::P*_spac_spoIIIJ*-*his_6_* Δ*yycR*::P*_sspE_-cfp* / Km^r^  Cm^r^ Erm^r^ | « |
| AH5432 | *trpC2 metC3* Δ*spoIIIJ*::*km* Δ*thrC*::P*_spac_spoIIIJ*^C134A^-*his_6_* Δ*yycR*::P*_sspE_-cfp* / Km^r^  Cm^r^ Erm^r^ | « |
| AH5433 | *trpC2 metC3* Δ*spoIIIJ*::*km* Δ*yycR*::P*_sspE_-cfp* / Km^r^  Cm^r^ | « |
| AH6566 | *trpC2 metC3* Δ*yycR*::P*_sspE_-cfp* | [25] |
| AH9218 | *trpC2 metC3* Δ*spoIIIJ*::*spoIIIJ-his_6_* / Sp^r^ | Côrte *et al*., manuscript in preparation ; [24] |
| AH9335 | *trpC2 metC3* Δ*spoIIAC*::*erm* Δ*yycR*::P*_sspE_-cfp* / Erm^r^  Cm^r^ | [25] |
| BTD2633 | Δ*yycR*::P*_sspE_-cfp* / Cm^r^ | D. Rudner |

^a^ Km, kanamycin; Cm, cloramphenicol; Erm, erythromycin; Sp, spectinomycin.
