# Supplementary material for: A Conserved Cysteine Residue of Bacillus subtilis SpoIIIJ Is Important for Endospore Development
Source: PLoS One. 2014 Aug 18;9(8):e99811. doi: 10.1371/journal.pone.0099811 (PMC4136701; doi:10.1371/journal.pone.0099811)
Supplement: Table S2 — Oligonucleotides used in this study. (DOC) [file pone.0099811.s006.doc]

**A conserved cysteine residue of *Bacillus subtilis* SpoIIIJ is important for endospore development**

Luísa Côrte**^1^**, Filipa Valente**^1*^**, Mónica Serrano**^1^**, Cláudio M. Gomes**^1^**, Charles P. Moran, Jr**^3^**., and Adriano O. Henriques**^1,3^**

Instituto de Tecnologia Química e Biológica, Universidade Nova de Lisboa,

Avenida da República, Apartado 127, 2oz781-901 Oeiras, Portugal**^1^**, and Department of Microbiology and Immunology, Emory University School of Medicine, Atlanta, Georgia 303222**^2^**

**Supporting information – Table S2**

**Table S2. Oligonucleotides used in this study.**

| **Primer** | Sequence (5´to 3´)^a^ |
| --- | --- |
| spoIIIJ112D | GGAGGCCATGGTGTTGAAAAGGAGAATAGGG |
| spoIIIJhis | GGAATTCTCAGTGGTGGTGGTGGTGGTGCTTTTTCTTTCCTCCGGCTTTTTGCGGC |
| spoIIIJC134A_D | GTCAATCCATTGGCGGGA**GCT**TTCCCGATTTTGATCCAG |
| spoIIIJC134A_R | CTGGATCAAAATCGGGAA**AGC**TCCCGCCAATGGATTGAC |
| spoIIIJ174D | GAGTGTAAAGATCTAATTATAGGAGG |
| spoIIIJhisR | ACATGCATGCTCA**GTGGTGGTGGTGGTGGTG**CTTTTTCTTTCCTCCGGCTTTTTGCGGC |
| YqjG-His-R | gaggatccTTTCACCGACTCAGTAAGAGCG |
| PYqjG-460D | AGAGCGGGATCCCTGTATGGTGTATCG |
| YA50C_D | gattatttaatcgaaccgttttcc**tgc**ctgcttaagggtgttgcc |
| YA50C_R | Ggcaacacccttaagcag**gca**ggaaaacggttcgattaaataatc |
| yqjGC142A_D | atcaacccgcttgcgatgggc**gct**cttccaatgctgattcagtctc |
| yqjGC142A_R | gagactgaatcagcattggaag**agc**gcccatcgcaagcgggttgat |

***^a^*** Restriction sites are underlined, mutations in bold.
