# Supplementary material for: A Conserved Cysteine Residue of Bacillus subtilis SpoIIIJ Is Important for Endospore Development
Source: PLoS One. 2014 Aug 18;9(8):e99811. doi: 10.1371/journal.pone.0099811 (PMC4136701; doi:10.1371/journal.pone.0099811)
Supplement: Table S3 — Plasmids. (DOC) [file pone.0099811.s007.doc]

**A conserved cysteine residue of *Bacillus subtilis* SpoIIIJ is important for endospore development**

Luísa Côrte**^1^**, Filipa Valente**^1*^**, Mónica Serrano**^1^**, Cláudio M. Gomes**^1^**, Charles P. Moran, Jr**^3^**., and Adriano O. Henriques**^1,3^**

Instituto de Tecnologia Química e Biológica, Universidade Nova de Lisboa,

Avenida da República, Apartado 127, 2oz781-901 Oeiras, Portugal**^1^**, and Department of Microbiology and Immunology, Emory University School of Medicine, Atlanta, Georgia 303222**^2^**

**Supporting information – Table S3**

**Table S3. Plasmids.**

| **Plasmid** | Relevant features | Origin/reference |
| --- | --- | --- |
| pFiV1 | pETDUET-1+*spoIIIJ*^C134A^-*his_6_* | This work |
| pFiV2 | pDH88+*spoIIIJ*-*his_6_* | « |
| pFiV3 | pDH88+*spoIIIJ*^C134A^-*his_6_* | « |
| pFiV4 | Δ*thrC*::P*_spac_-spoIIIJ-his_6_* | « |
| pFiV5 | Δ*thrC*::P*_spac_-spoIIIJ*^C134A^*-his_6_* | « |
| pMS266 | pETDUET-1+*spoIIIJ*-*his_6_* | [25] |
| pLC111 | Δ*amyE*::*his_6_* | Côrte *et al*., manuscript in preparation |
| pLC115 | Δ*amyE*::*yqjG-his_6_* | Côrte *et al*., manuscript in preparation |
| pLC138 | Δ*amyE*::*yqjG*^A50C^-*his_6_* | This work |
| pLC155 | Δ*amyE*::*yqjG*^A50C/C142A^-*his_6_* | « |
| pDH88 | P*_spac_* | [31] |
| pDG1664 | *thrC* insertion | [32] |
